# The *Arabidopsis thaliana* Immunophilin ROF1 Directly Interacts with PI(3)P and PI(3,5)P_2_ and Affects Germination under Osmotic Stress

**DOI:** 10.1371/journal.pone.0048241

**Published:** 2012-11-02

**Authors:** Debora Karali, David Oxley, John Runions, Nicholas Ktistakis, Theodora Farmaki

**Affiliations:** 1 Institute of Applied Biosciences, Centre for Research and Technology – Hellas, Thermi, Thessaloniki, Greece; 2 The Mass Spectrometry Group, Babraham Institute, Cambridge, United Kingdom; 3 School of Life Sciences, Oxford Brookes University, Oxford, United Kingdom; 4 Signalling Programme, Babraham Institute, Cambridge, United Kingdom; Purdue University, United States of America

## Abstract

A direct interaction of the *Arabidopsis thaliana* immunophilin ROF1 with phosphatidylinositol-3-phosphate and phosphatidylinositol-3,5-bisphosphate was identified using a phosphatidylinositol-phosphate affinity chromatography of cell suspension extracts, combined with a mass spectrometry (nano LC ESI-MS/MS) analysis. The first FK506 binding domain was shown sufficient to bind to both phosphatidylinositol-phosphate stereoisomers. GFP-tagged ROF1 under the control of a 35S promoter was localised in the cytoplasm and the cell periphery of *Nicotiana tabacum* leaf explants. Immunofluorescence microscopy of *Arabidopsis thaliana* root tips verified its cytoplasmic localization and membrane association and showed ROF1 localization in the elongation zone which was expanded to the meristematic zone in plants grown on high salt media. Endogenous ROF1 was shown to accumulate in response to high salt treatment in *Arabidopsis thaliana* young leaves as well as in seedlings germinated on high salt media (0.15 and 0.2 M NaCl) at both an mRNA and protein level. Plants over-expressing ROF1, (WSROF1OE), exhibited enhanced germination under salinity stress which was significantly reduced in the *rof1^−^* knock out mutants and abolished in the double mutants of ROF1 and of its interacting homologue ROF2 (WS*rof1^−^/2^−^*). Our results show that ROF1 plays an important role in the osmotic/salt stress responses of germinating *Arabidopsis thaliana* seedlings and suggest its involvement in salinity stress responses through a phosphatidylinositol-phosphate related protein quality control pathway.

## Introduction

Phosphatidylinositol phosphates (PIPs) are phospholipids with important regulatory properties in signaling and trafficking processes [1]. PIPs possess a characteristic subcellular localization pattern [2], and the position of the phosphate group(s) on the inositol ring determines the function of these secondary messengers in several cellular events through their recognition by specific phosphoinositide binding domains [3]. PIPs’ ability to transmit and communicate changes arising in the lipid bilayer as a result of a biochemically transmitted environmental signal has been extensively described in many eukaryotic systems. Although not all PIP stereoisomers identified and functionally characterized in mammalian systems exist in plants, PIPs with important regulatory role in stress and development have been described in plant systems [4]. Their importance during these processes becomes evident through the diverse effects that the absence of a PIP-related kinase or phosphatase may convey to the system. Their abrogation involves severe effects on growth and development, morphological distortion in both subcellular and tissue level, alterations in cell organelle organization and structure and disturbance of hormonal levels and sensitivity [4]. For the PIP steroisomer phosphatidylinositol-3,5-bisphosphate [PI(3,5)P_2_], phenotypic effects of its absence have been described in yeast, however, in plants our knowledge is limited. Absence of Fab1p, the only kinase that synthesizes PI(3,5)P_2_ in yeast through a direct modification which converts phosphatidylinositol-3-phosphate [PI(3)P] to PI(3,5)P_2_, results in enlarged vacuoles [5], [6], [7]. In yeast PI(3,5)P_2_ is increased following hyperosmotic stress which also accelerates the PI(3,5)P_2_ mediated fission of lysosomal subcompartments [8]. In plants both PI(3)P and PI(3,5)P_2_ have been implicated in osmotic stress responses [9], [10], [11], [12]. However, there is no evidence of a PI(3,5)P_2_ related response in *A. thaliana* seedlings or suspension cultures [13]. The small amount of PI(3,5)P_2_ contained in plants is increased in response to osmotic stress, however, our knowledge of the functional importance of PI(3,5)P_2_ on plant osmotic stress responses is very limited. In addition, PI(3,5)P_2_ presents a peculiarity compared to other PIPs. In contrast to the PI(3)P, phosphatidylinositol-4,5-bisphosphate [PI(4,5)P_2_] and phosphatidylinositol-3,4,5-trisphosphate [PI(3,4,5)P_3_], PI(3,5)P_2_ recognizing domains are highly diverse; e.g. HEAT/ARM repeat containing ENTH domains [14], [15], a polybasic N-terminus [16], a PX domain [17], [18], [19] or a beta-propeler structure may be utilized for binding [20]. Because of our limited knowledge of the role of PI(3,5)P_2_ during osmotic stress responses in plants and a still unidentified PI(3,5)P2 effector, the identification of novel PI(3,5)P_2_ binding candidates from osmotically stressed *A. thaliana* extracts would provide useful information on both the functional importance of PI(3,5)P_2_ in plants as well as for novel binding domains interacting with this phosphoinositide.

In this work we show that ROF1, a plant immunophilin that belongs to the FK506-Binding Protein (FKBP) subfamily, directly interacts with PI(3)P and PI(3,5)P_2_ and is involved in plant responses to high salinity and osmotic stress. Following its isolation using a phosphatidylinositol-phosphate (PIP) affinity chromatography its mode of interaction with PIP stereoisomers was investigated as well as the possible functional importance of this interaction in cellular processes during plant osmotic stress responses.

## Results

### FKBP Purification Using an Agarose Coupled PIP Affinity Chromatography

**Figure 1:**
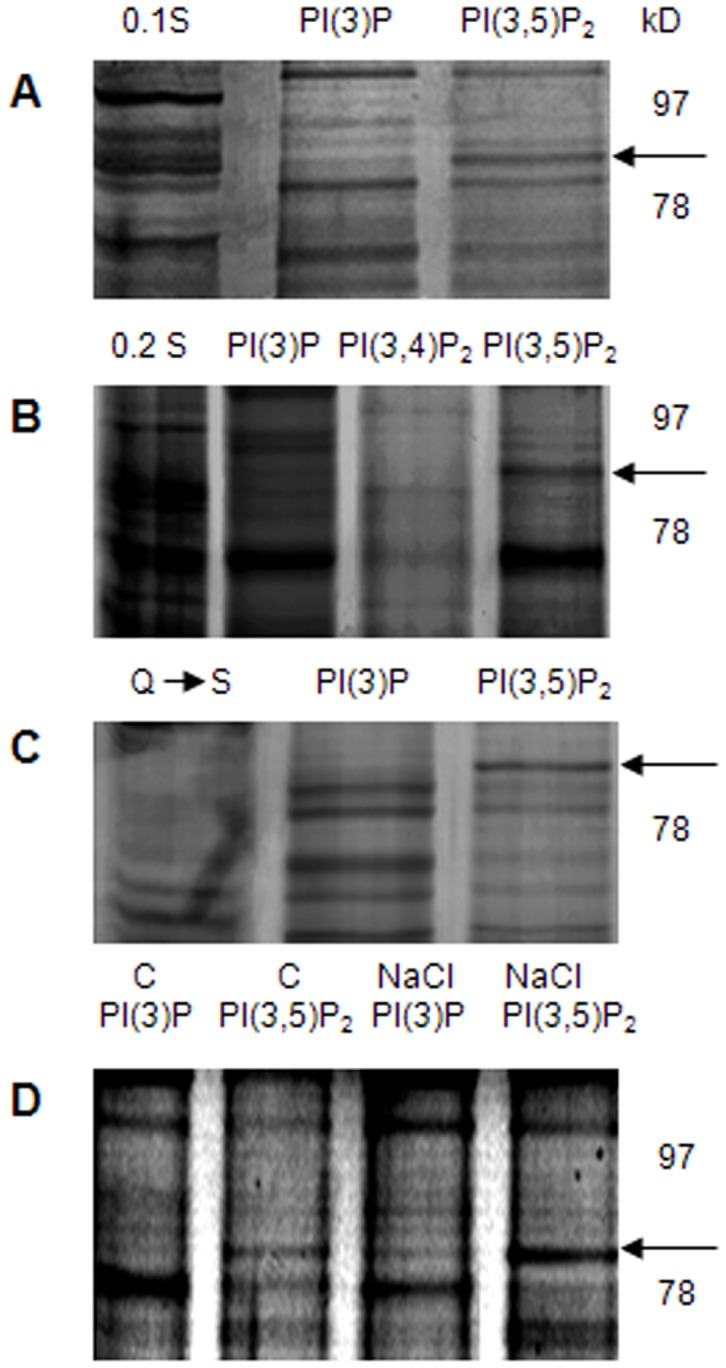
Identification of a band enriched in a PI(3,5)P_2_ affinity chromatography of *A. thaliana* cell extracts, run on an SDS-PAGE and silver stained. (A) 0.1S (0.1M NaCl elution of the S ion-exchange chromatography; see materials and methods) fraction of *A. thaliana* extract incubated with the PI(3)P and PI(3,5)P_2_ columns. (B) 0.2S fraction incubated with PI(3)P, PI(3,4)P_2_ and PI(3,5)P_2_ columns. (C) FT collected after sequential incubation with a Q followed by an S column (Q–>S) and applied to a PI(3)P and PI(3,5)P_2_ affinity chromatography. (D) Incubation of a 0.2S fraction -obtained from extracts of 0 M (C) or 0.4 M NaCl treated, culture cells - with PI(3)P and PI(3,5)P_2_ columns_._ Arrows indicate an enriched band in the PI(3,5)P_2_ incubation (A, B and C) as well as its accumulation in both the PI(3)P and PI(3,5)P_2_ incubations following salt stress of the *A. thaliana* cell culture (D).

A PIP chromatography was employed in order to identify PI(3)P and PI(3,5)P_2_ binding candidates from *A. thaliana* cell suspension cultures. A T87 cell suspension culture treated with either 0 (C) or 0.4M NaCl for 5 mins was centrifuged, cell pellet was collected and homogenized and extracts were first incubated with a positively and a negatively charged ion exchange chromatography (Q and S Sepharose) in order to reduce sample complexity. Sequential washings using different salt concentrations (see materials and methods) resulted in fractions which in turn were incubated with the PIP chromatography. A band close to 80 kD apparent molecular weight appeared enriched in the PI(3,5)P_2_ incubation of several Q and S fractions (Figure 1 A–C). Salt stress (0.4 M NaCl) for 5 mins resulted in its enrichment in both the PI(3)P and PI(3,5)P_2_ incubations of the 0.2 M Q fraction (Figure 1D). Band excision from both lanes of the PI(3)P and PI(3,5)P_2_ incubation of the salt stressed cultures (Figure 1D), followed by nanoLC ESI-MS/MS identified the peptidyl-prolyl cis-trans isomerases (PPIases) ROF1 (Q9LSF3; predicted MW: 63 kD; Mascot score for PI(3)P: 209; 4 peptides used with 100% probability and Mascot score for PI(3,5)P_2_∶730; 13 peptides used with 100% probability) and ROF2 (Q9FJL3; predicted MW: 65 kD; Mascot score for PI(3,5)P_2_∶427; 8 peptides used with 100% probability) (see Supplementary data; Spectrum Report S1).

### Structural Prediction for ROF1

**Figure 2:**
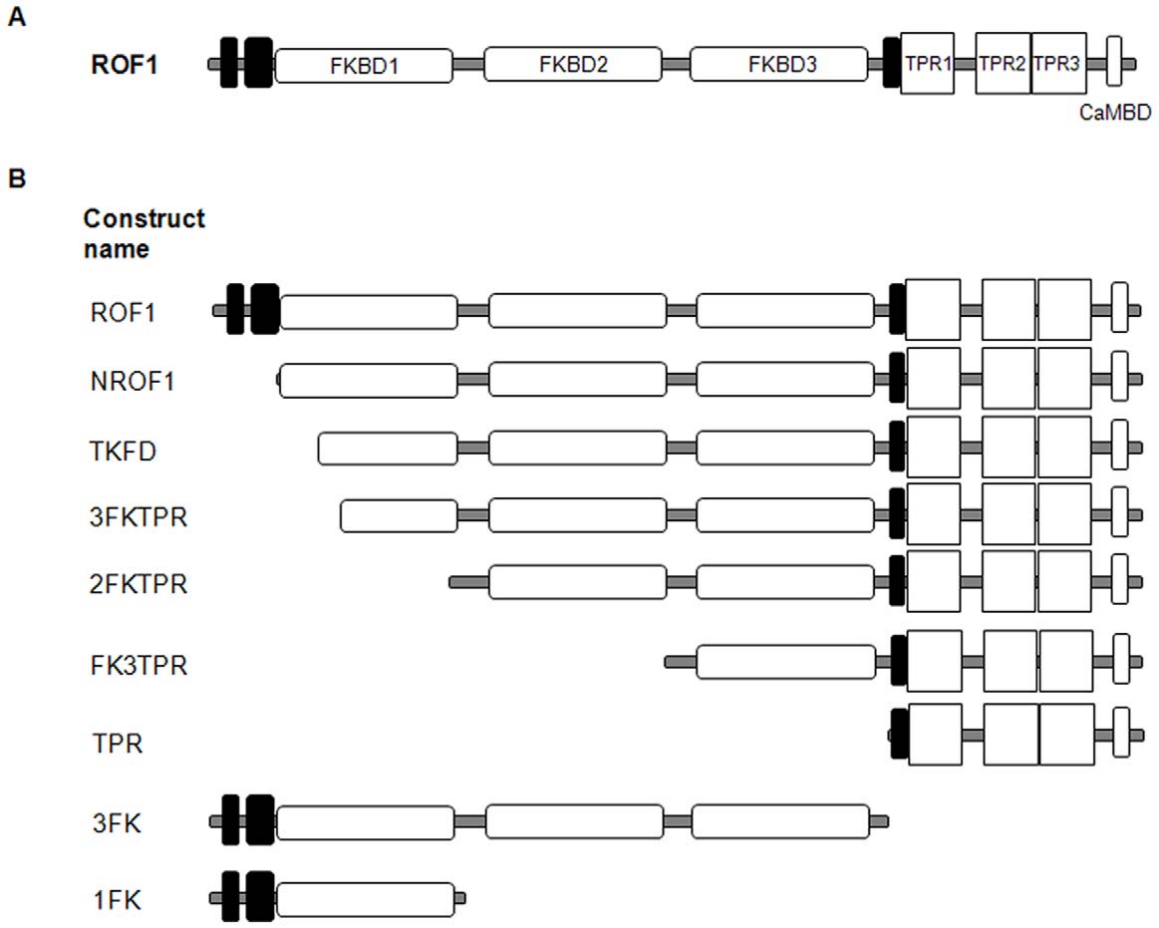
ROF1 construct description. (A) Domain organization of ROF1. Black shaded boxes indicate low complexity regions (source: PFAM and UNIPROT). (B) ROF1 and ROF1 truncated mutants used in GST-His fusions for protein overexpression. NROF1: truncated ROF1 missing the first 37 aminoacids of the protein (low complexity region) (starting aminoacids: QGLKKKLL). TKFD: truncated ROF1 missing the low complexity region part of the FKBD1 and the polylysine motif (starting aminoacids: TKFDSSR). 3FKTPR: truncated ROF1 missing the N- terminus sequence containing both the polylysin and the DSSRDR motives (starting aminoacids: PFKFTLG). Also see Figure S1.

ROF1 is a multi-domain protein. ROF1 contains an FK506 Binding Domain (FKBD) with PPIase activity, (FKBD1), two degenerate FKBDs (FKBD2 and FKBD3) as well as tetratricopeptide repeat domains (TPR) and a potential calmodulin binding domain [21] (Figure 2A; Figure S1). The PDB structure of the structural homologue of the FKBP12, β-spectrin, was obtained (http://www.ncbi.nlm.nih.gov/structure) known for binding to the I(1,4,5)P_3_ [22], [23], and the residues binding to the I(1,4,5)P_3_ were identified (Figure S2). In particular, the structural studies have indicated that β-spectrin specifically interacts with I(1,4,5)P_3_ at K8, R21, S22, W23, Y69 and K71. Also, the 3D structures of the FKBD domains of ROF1 were obtained (http://swissmodel.expasy.org) and aligned to β-spectrin (Figure S2).

Structural alignment of β-spectrin and the FKBD1 domain of ROF1 using DALI pair- wise comparison (http://ekhidna.biocenter.helsinki.fi/dali_lite/start) and Cn3D (http://www.ncbi.nlm.nih.gov/Structure/CN3D/cn3d.shtml) indicated possible binding sites for a phosphorylated inositol ring on the FKBD1 domain of ROF1 (Figure S2) involving the DSSRDR region.

### Bacterially Expressed ROF1 Interacts with PI(3)P and PI(3,5)P_2_ through its FKBD Domains

**Figure 3:**
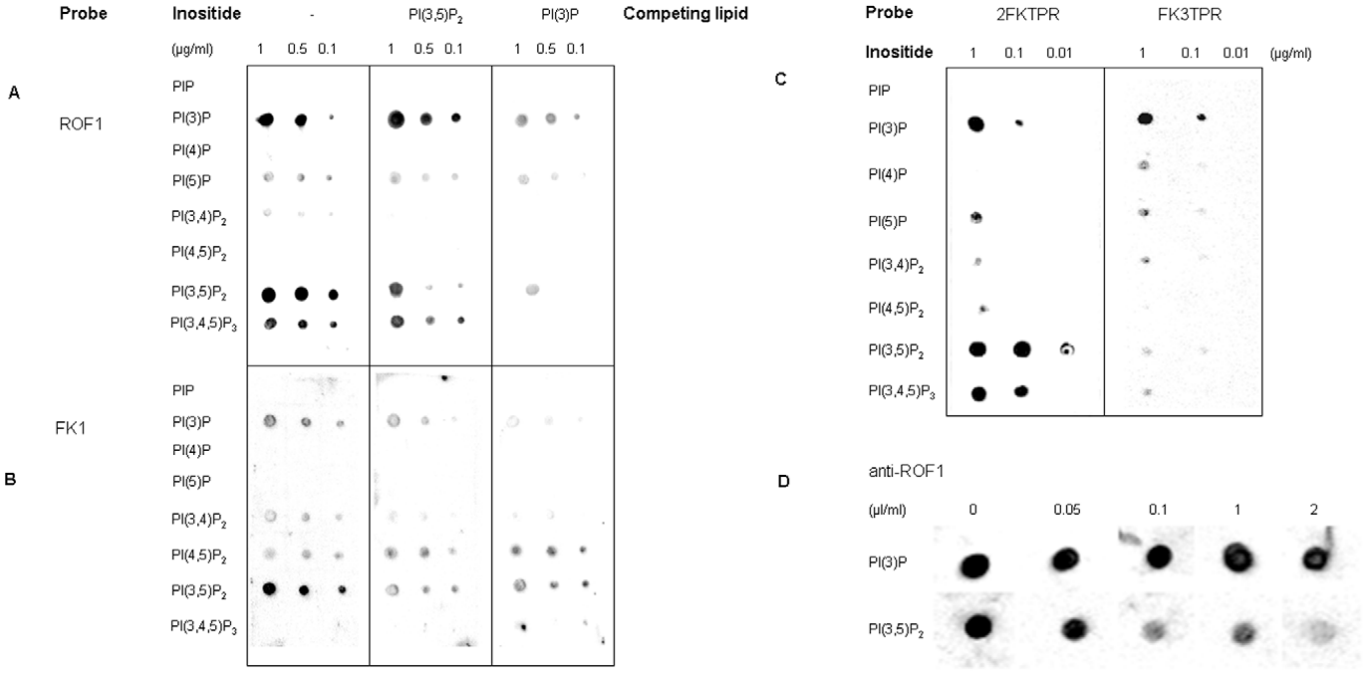
Lipid overlay assays in order to characterize ROF1 binding to different PIP stereoisomers. Dot blotting of different PIP stereoisomers probed with 1 µg/ml of the ROF1 protein (A) and its truncated mutant 1FK (FKBD1) (B) (Figure 2B; Figure S1) and detected with αντι-GST. (−): No competing lipid has been added in the incubation, [PI(3,5)P_2_]: 50 µM of the lipid has been added during the incubation; [PI(3)P]: 50 µM of the lipid has been added during the incubation. (C) Dot blotting of different PIP stereoisomers probed with 2 µg/ml of the ROF1 truncated mutants 2FKTPR and FK3TPR (Figure 2B; Figure S1) and detected with αντι-GST. (D) Characterization of the binding site of ROF1 to PI(3)P and PI(3,5)P_2_ using the N-terminus specific antibody anti-ROF1. Overexpressed ROF1 was pre-incubated with different concentrations of the antibody, as indicated, prior to its use in a lipid overlay assay and detected with anti-GST.

ROF1 and its truncated mutants (Figure 2B; Table S1; Table S2) were over-expressed with an N-terminal GST tag and C terminal-His tag following transformation of a BL21 bacterial cell line with the pALEX vector used for their cloning. Following double purification using Glutathione and Nickel columns ROF1 and its truncated mutants were eluted and used in a lipid overlay assay (Figure 3). ROF1 specifically associated with both the PI(3)P as well as with the PI(3,5)P_2_ (Figure 3A). The FKBD1 domain was shown sufficient for PIP binding and appeared to possess a higher affinity for the PI(3,5)P_2_ stereoisomer and reduced affinity for the PI(3)P (Figure 3A, B). A competition assay, using the two stereoisomers as competing lipids, produced a specific reduction in the case of the PI(3,5)P_2_, however, an addition of PI(3)P as a competing lipid reduced signal for both the PI(3)P and the PI(3,5)P_2_ in the ROF1 and the FKBD1 assay (Figure 3A, B). Although the FKBD1 was shown to possess a high affinity for the PI(3,5)P_2_ its absence did not abolish binding to the PI(3,5)P_2_ stereoisomer as demonstrated by the ability of the 2FKTPR construct to interact with both lipids (Figure 3C). On the other hand, FK3TPR, only interacted with the PI(3)P (Figure 3C). Although the TPR-calmodulin binding domain alone was also over-expressed, we were unable to solubilise it and the entire amount of protein remained inside the inclusion bodies. The results suggest a differential capacity of the FKBD domains for lipid binding as well as the ability of ROF1 to bind to PI(3,5)P_2_ in the absence of the FKBD1 inspite the fact that FKBD2 and FKBD3 do not possess a structural identity to β-spectrin and predictable PIP binding sites, at least not to the same extent as the FKBD1 does (Figure S2).

### Use of the Αντι-ROF1 for the Characterization of the PI(3,5)P_2_ Binding Site

A polyclonal antibody was raised against the bacterially expressed ROF1 as described in the Materials and Methods. Taking into account that αντι-ROF1 was raised against the denatured protein, ROF1 truncated mutants (Figure 2B) expressed in BL21 cells were purified and run on a polyacrylamide gel. Western blotting using αντι-GST as a control, in order to confirm that the entire and correct insert was expressed, showed that αντι-ROF1 specifically recognizes the N-terminus of the protein (Figure S3). In particular, the antibody showed no affinity for the TPR-calmodulin domain as well as for the FKBD2 and the FKBD3 (Figure 2B; Figure S1; Figure S3A). It also showed a highly reduced affinity for the FKBD1 domain not containing the N-terminal residues prior to the DSSRDR sequence (TKFD construct) (Figure 2B; Figure S1; Figure S3B). The antibody showed a complete recognizing ability for the ROF1 providing that the polylysine rich patch (KKKLLK) was present (NROF1 construct) (Figure 2B; Figure S1; Figure S3C). Taking into account the specific site of antibody recognition, we used the antibody in order to block ROF1 prior to the incubation in the lipid overlay assay. Different concentrations of the ROF1 antibody were used in a mixture with the protein (0 to 2 µl/ml) and following incubation in a lipid overlay assay as previously described, αντι-GST was used for the detection of protein-lipid interaction (Figure 3D). The antibody pre-incubation reduced binding affinity for the 3,5 phosphorylated lipid but not for the lipid phosphorylated at the third position of the inositol ring (Figure 3D).

**Figure 4:**
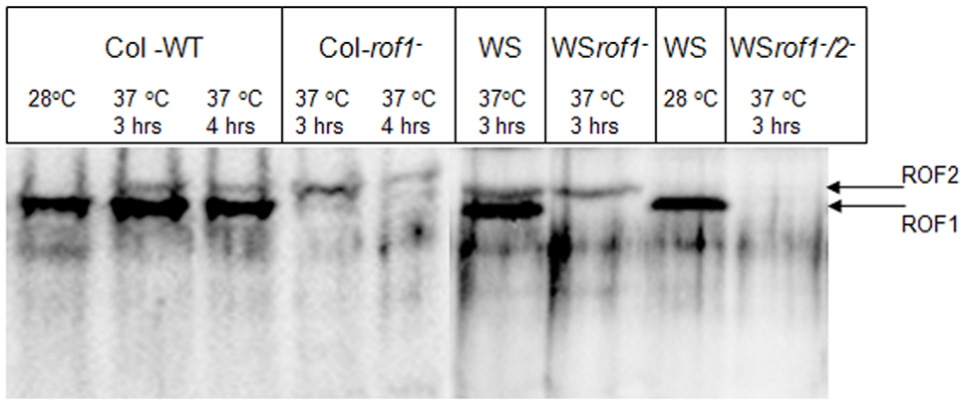
Recognition of ROF1 and ROF2 using anti-ROF1. 5-day old *A. thaliana* wild type, ROF1 knock-out (*rof1^−^*) of both Columbia and Wassilevskija background and ROF1/2 double mutants (WS*rof1^−^/2^−^*) were incubated at 37°C for up to 4 hrs. anti-ROF1 specifically detects the ROF1 protein in plant extracts and to a less extent a second upper band (ROF2) following heat treatment only. Columbia ROF1 knock-out belongs to the WDsLox line.

Antibody specificity was further verified using both *A. thaliana* wild type and mutant lines (WDsLOX, WS*rof1^−^*, WS*rof1^−^/2^−^*) stressed at 37°C for up to 4 hrs. Results showed (Figure 4; Figure S4) that the αντι-ROF1 specifically recognizes endogenous ROF1 and to a very low extent its homologous partner ROF2 providing the latter has been accumulated following heat stress [24] (Figure 4).

### ROF1 is Induced Under Salinity Stress

**Figure 5:**
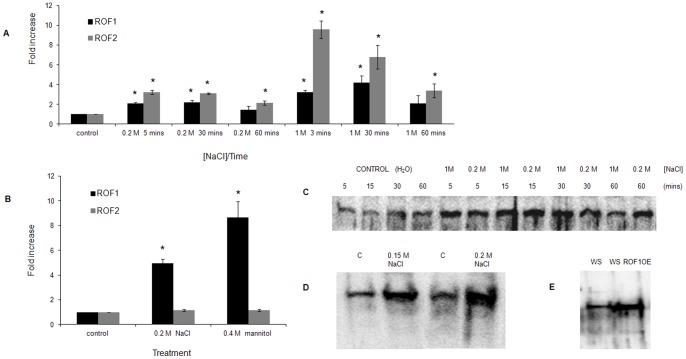
Gene expression and protein accumulation study for ROF1. (A) Real time PCR of wild type *A. thaliana* plants treated for different time points and under different salt concentrations (0 M, 0.2M and 1M NaCl). Results are expressed as fold increase relative to the control set at 1. Control represents mock treatment with H_2_O for the respective time points to the NaCl treatment. (B) Real time PCR of control (C) wild type 4-day old *A. thaliana* seedlings germinated on MS media and of seedlings germinated and grown on media containing NaCl or mannitol. Results are expressed as fold increase relative to the control set at 1. Control represents plants grown on MS media. Results are average of three independent experiments performed in triplicates. (C) Western blotting of wild type *A. thaliana* young leaves obtained from plants which have been treated for different time points and at different salt concentrations (0 M, 0.2M and 1M NaCl). Control represents mock treatment with H_2_O for the respective time points to the NaCl treatment. (D) Western blotting of control (C) wild type 4 day old *A. thaliana* seedlings germinated on MS media and seedlings germinated and grown on MS media containing different NaCl concentrations. (E) Western blotting of WS and WSROF1OE seeds 3 hrs after water imbibition.

3 week old *A. thaliana* plants were incubated with 0, 0.2 or 1 M NaCl. In young leaves collected at different time points, ROF1 and ROF2 accumulation was observed at an mRNA level following salt treatment (Figure 5A). A fast induction within 3 mins appeared following incubation with 1 M NaCl for both ROF homologues. mRNA accumulation was also observed in the case of ROF1 in seedlings germinated on high salt/osmotic media compared to the control (MS media). However, this accumulation was not observed for ROF2 (Figure 5B). ROF1 accumulation was also observed at a protein level (Figure 5C). No apparent differences were, however, observed in ROF1 protein accumulation at high salt concentrations (1 M) compared to lower salt concentrations (0.2 M) but an earlier reduction was observed for the 1 hr 1 M NaCl treatment compared to the 0.2 M incubation. Protein accumulation was also observed in the case of whole seedlings, germinated in high salt MS media (0.15 and 0.2 M NaCl) compared to the control seedlings (Figure 5D). The presence of ROF1 was also confirmed in non-germinated seeds only 3 hrs after water imbibition (Figure 5E).

### Over-expressed and Endogenous ROF1 Localization Under Control and Osmotic/salinity Stress Conditions

**Figure 6:**
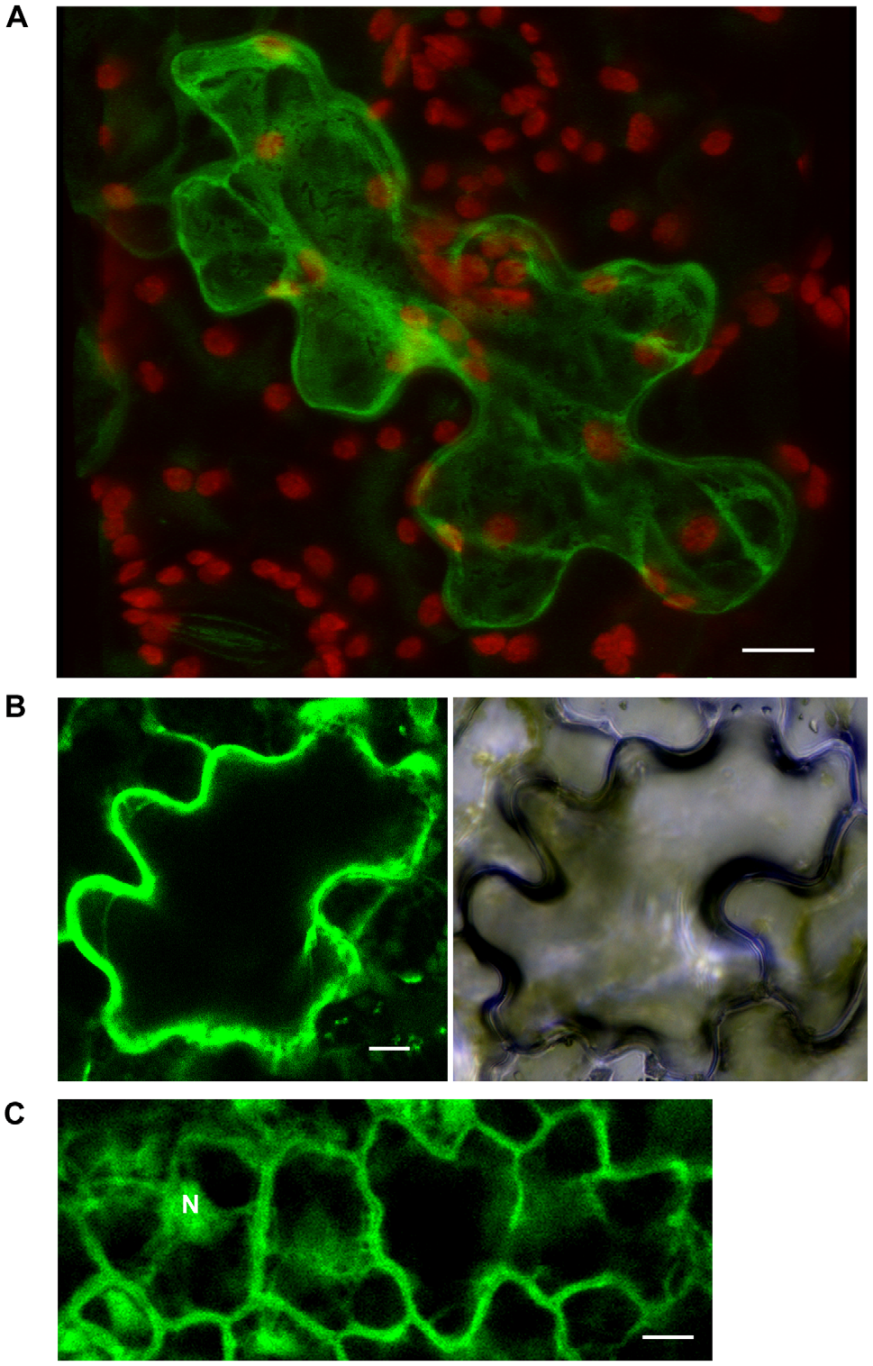
ROF1 localisation using tobacco plants transiently transformed and expressing GFP tagged ROF1 and GFP only. (A) Control (z-stack image projection), green: ROF1-GFP, red: chloroplast autofluorescence. (B) GFP-tagged ROF1 localization and bright field imaging (single confocal section) (C) GFP localization (single confocal section). N indicates nuclei. Scale bars: 10 µm.

Using an *A. tumefaciens* plant expression vector we transiently transformed tobacco leaves with constructs containing a full length ROF1. A cytoplasmic localization of the protein was observed using a 3D reconstruction (Figure 6A) as demonstrated by the traces (empty spaces) that the embedded organelles mark in the cytoplasm and the cytoplasmic strands formed. A peripheral localization may also be observed, more clearly seen in a single confocal section (Figure 6B). ROF1 localization was distinct to GFP localization since no nuclear localization of the GFP tagged-ROF1 was observed (Figure 6C). In order to confirm the nature of the peripheral localization of ROF1, a plasmolysis effect caused by 0.25 M NaCl treatment was used. As it is shown (movie S1), the peripheral staining of ROF1 is not due to a protein association with the cell wall.

**Figure 7:**
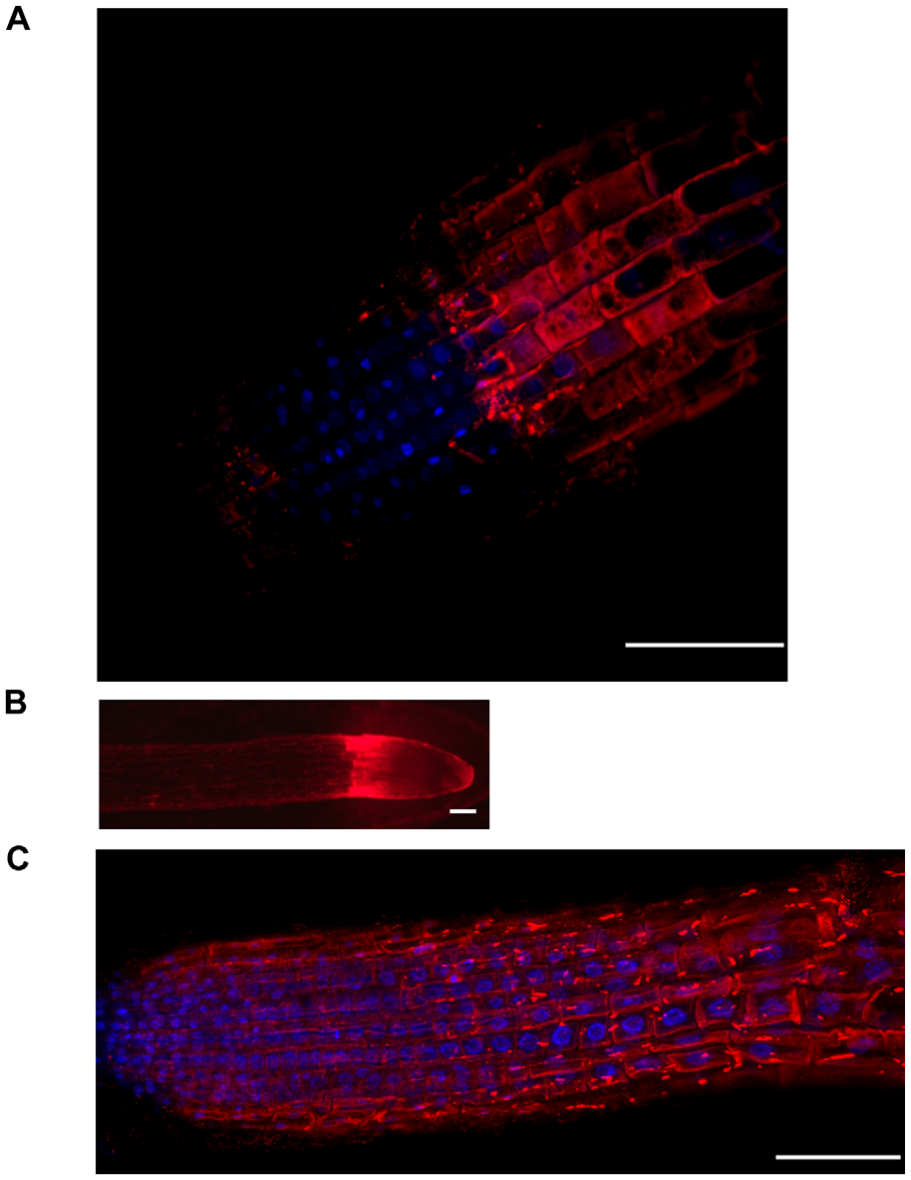
Localization of ROF1 in 4% PFA fixed *A. thaliana* root tips of 4 day old plants using αντι-ROF1. (A) confocal image (z-stack projection) of untreated (control) plants; red: ROF1, blue: DAPI. (B) fluorescence microscope image of plants treated with 0.4 M NaCl for 2 mins. (C) confocal image (z-stack projection) of plants grown in 0.2 M NaCl; red: ROF1, blue: DAPI. Scale bars: 50 µm.

In order to further verify ROF1 subcellular localization immunofluorescence microscopy with *A. thaliana* root tips was used. *A. thaliana* seedlings were germinated on 0.8% agar media containing 1×MS and immunofluorescence microscopy was performed as described in the Materials and Methods. Incubation with pre-immune serum or αντι-ROF1 staining of *rof1^−^* plants did not produce any fluorescence (results not shown) confirming antibody specificity as described above. In untreated plants and plants treated for a short period of time with high NaCl (0.4 M for 2 mins) ROF1 was mainly localized in the elongation zone of the root tip (seedlings were immersed in the solution washed briefly and then fixed) (Figure 7A, B; Movie S2). However, in plants grown on high salt media (0.2 M NaCl), the localization pattern changed, expanding throughout the meristematic zone (Figure 7C; Movie S3). The cytoplasmic localization of ROF1 was confirmed with immunofluorescence microscopy (Figure 7A; Movie S2) whereas a stronger peripheral staining was observed in plants grown on high salt media (Figure 7C; Movie S3) probably indicating membrane association. In any case, no nuclear localization was observed.

### Effect of Osmotic/ionic Stress on the Germination of Wild Type and ROF Mutant *A. thaliana* Seeds

**Figure 8:**
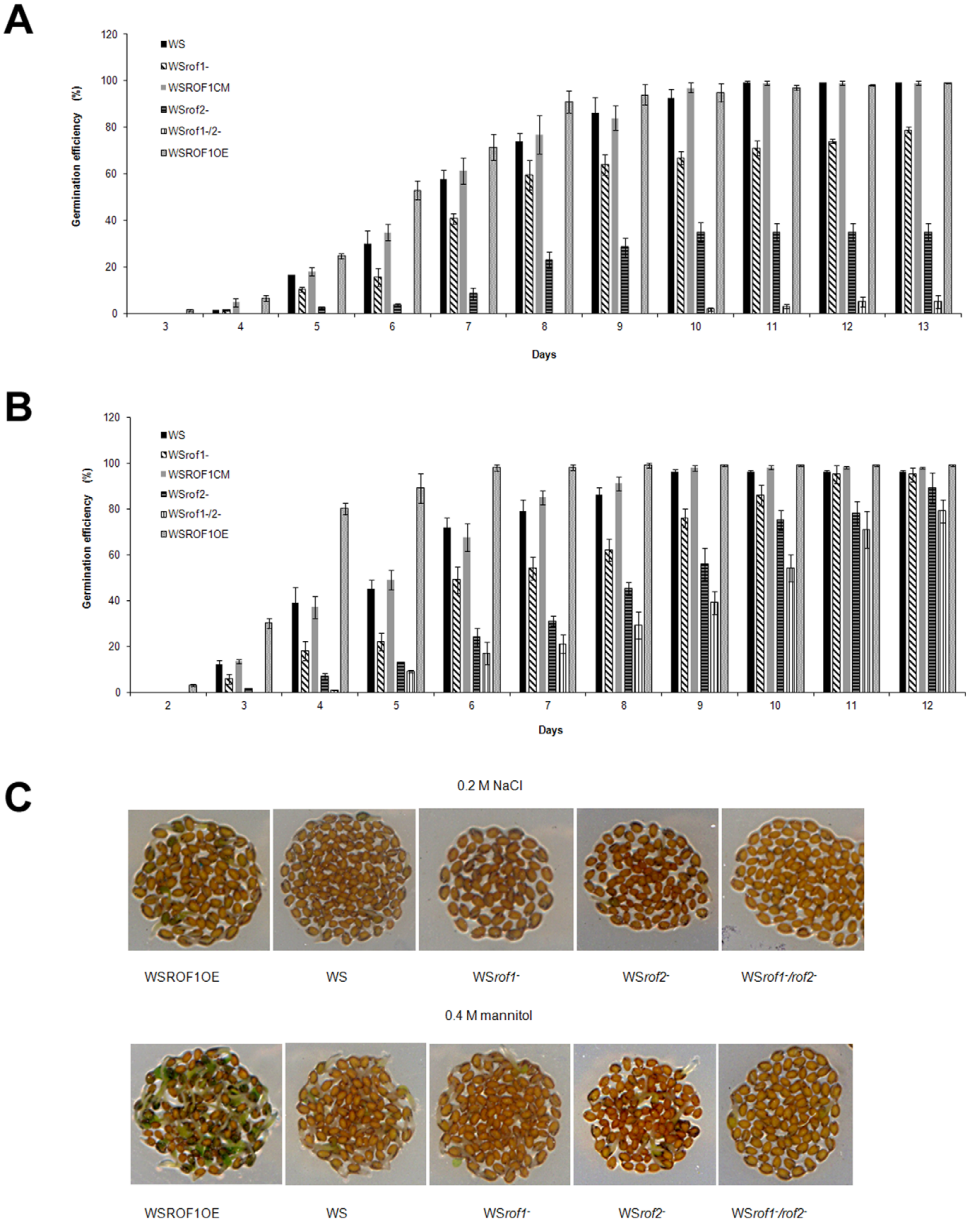
Germination efficiency of ROF1 and ROF2 mutants under osmotic/salinity stress. (A) Germination rate of the Wassilevskija background plants WS, WS*rof1^−^*, WS*rof2^−^*, WS*rof1^−^/2^−^*, WSROF1CM and WSROF1OE on MS media containing 0.2 M NaCl. (B) Germination rate of the wild type Wassilevskija background plants WS, WS*rof1^−^*, WS*rof2^−^*, WS*rof1^−^/2^−^*, and WSROF1OE on MS media containing 0.4 M mannitol. Three independent characterized lines (in each experiment) were used for each genotype. The results show the average of three independent experiments performed for each treatment. Values are means, and bars are SDs. (C) Seeds germinated on MS media containing 0.2 M NaCl or 0.4 M mannitol (day 6, 22°C).

Several lines of Columbia wild type (Col-WT), Columbia *rof1^−^* (Col- *rof1^−^*), Wassilevskija wild type (WS), WS*rof1^−^* and WS*rof1^−^*/*rof2^−^*, WS complemented mutants (WSROF1CM) and WS ROF1 over-expressions (WSROF1OE) were confirmed by western blotting using the αντι-ROF1 for ROF1 detection (Figure 4; Figure S4). Columbia WT as well as *rof1^−^* seeds on a Columbia background were germinated on media containing different NaCl concentrations. Although no difference was recorded on the germination rate of plants grown on media without NaCl in relation to the WT (Figure S5A, D) a significant delay was recorded on the germination of *rof1^−^* plants germinated on media containing salt concentrations of 0.2 M NaCl (Figure S5B). The NaCl effect on plant germination was also observed with the Wassilevskija wild type and *rof1^−^* (Figure 8A). Germination was nearly eliminated at 0.2 M NaCl when the WS*rof1^−^*/*2^−^* double mutants were used (Figure 8A, C). In order to show that the osmotic effect of ROF1 is exclusively due to the loss of the ROF1 gene complemented WS*rof1^−^* as well as WSROF1OE lines were used. It was shown that gene complementation completely recovered the wild type phenotype whereas gene over-expression resulted in a highly enhanced germination under salinity stress (Figure 8A). In order to differentiate between the ionic and the osmotic effect, WS, WSROF1OE, WS*rof1^−^*, WSROF1CM, WS*rof2^−^* and WS*rof1^−^*/*2^−^* were geminated on media containing 0.4 M mannitol and the osmotic effect on plant germination was confirmed (Figure 8B, C; Figure S5C).

### ROF1 Affects *A. thaliana* Seed Germination through a Phosphatidyl-Inositol-3 Kinase (PI3K) Related Pathway

**Figure 9:**
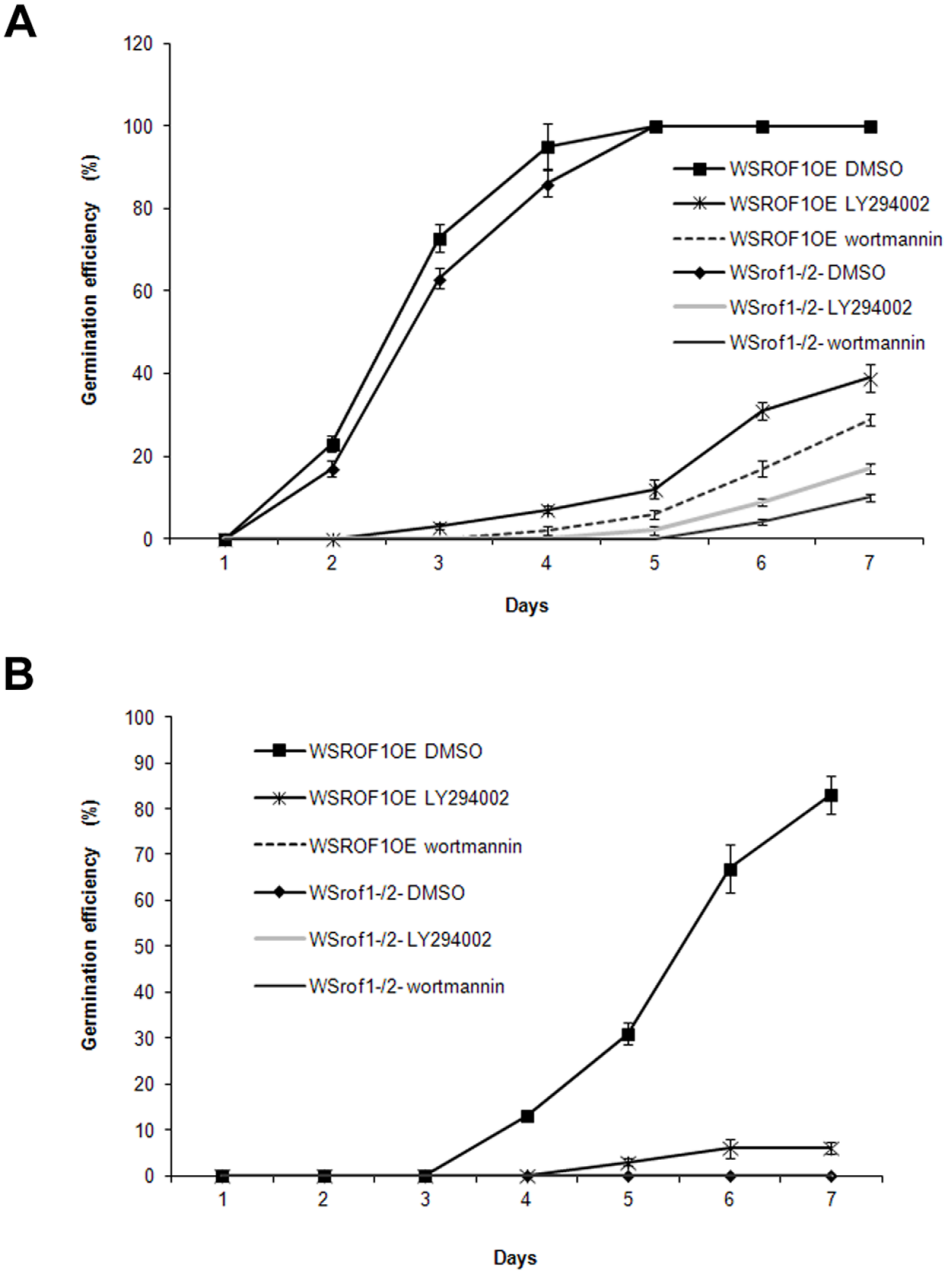
Effect of PI3K inhibitors on seed germination. Seed germination efficiency of WSROF1OE and WS*rof1^−^/2^−^* at different time points under control (A) or salinity stress (0.1 M NaCl) (B) in the presence or absence of 33 µM wortmannin or 60 µM LY294002. The results show the average of four independent experiments performed for each treatment. Values are means and bars are SDs.

*A. thaliana* WSROF1OE and WS*rof1^−^/2^−^* seeds were germinated in the presence or absence of wortmannin or LY294002 under both control and salinity stress conditions. Germination was performed in darkness due to the photosensitivity of the wortmannin and LY294002. The NaCl concentration was 0.1 M instead of 0.2 M, used in previous experiments, due to the fact that germination efficiency in darkness in the presence of high salt concentrations was severely affected. Our results demonstrate that the PI3K inhibitors significantly affected seed germination of the double mutants under non-stress conditions (Figure 9A). In contrast, the germination efficiency of the WSROF1OE seeds was severely affected in the presence of the PI3K inhibitors when plants were germinated on 0.1 M NaCl, and in the case of wortmannin treatment WSROF1OE exhibited the same phenotype as the WS*rof1^−^/2^−^* plants under salinity stress i.e. their germination was completely abolished (Figure 9B).

## Discussion

Our work reports on the direct interaction of the plant FKBP ROF1 with PI(3)P and PI(3,5)P_2_ revealed by a PIP chromatography and a lipid overlay assay and investigates the physiological importance of this interaction.

FKBPs - immunophilins with PPIase activity [25], [26] - named after their property to bind the immunosupresant drug FK506 have been extensively studied in both mammalian and plant systems [27], [28], [29]. A remarkable characteristic of the FKBPs is their occurrence in every genome – from prokaryotes to higher eukaryotes- and in every part of the cell besides their occurrence as both single and multi-domain proteins as well as their involvement in a wide range of cellular processes [30]. Plant FKBPs are involved in several stress responses and developmental processes [31], [32], [33], [34], [35]. ROF1, a multi-domain high MW FKBP [36] (Figure 2; Figure S1), has already been shown to interact with Heat Shock Proteins (HSPs) and be temperature regulated [24], [37]. Its involvement in thermotolerance together with its interacting partner ROF2 has also been suggested [37], [38].

In this study, ROF1 and ROF2 were identified as PI(3)P and PI(3,5)P_2_ binding candidates in a differential screening approach which was followed in order to isolate novel binding proteins with a preference for the PI(3,5)P_2_ [compared to the PI(3)P] aiming at the identification of a PI(3,5)P_2_ binding domain (Figure 1 and Spectrum Report S1). The PI(3,4)P_2_ was also used as a negative control [no PI(3,4)P_2_ has been identified in plants] and no band enrichment was observed in this case (Figure 1B). ROF1 contains a poorly ordered domain that possibly results in its abnormal migration in the gel (predicted molecular weight: 63 kD, apparent MW about 80 kD) [21], [39].

There is only one extended study of immunophilin association with soluble inositol phosphates and PIPs. A protein called IPBP12 [40], [41] was reported to be strongly inhibited by nanomolar concentrations of inositol 1,4,5-trisphosphate (IP_3_), inositol 1,3,4,5-tetrakisphosphate (IP_4_), and phosphatidylinositol 4- and 4,5-phosphates. Both PIPs were suggested – by the same study- as potent ligands of this protein. It has also been reported that β-spectrin, an I(1,4,5)P_3_ binding protein, shows structural homology with FKBP12 [22], [23] a fact also predicted for the FKBD domains of ROF1 following structural alignment with β-spectrin (Figure S2). Our study suggests that ROF1 specifically interacts with PI(3)P and PI(3,5)P_2_ through its FKBD domains. It was also shown that the TPR/calmodulin binding domain is not necessary for binding to the PI(3)P and PI(3,5)P_2_ (Figure 3). The truncated mutant and the antibody blocking studies (Figure 2; Figure 3) indicate a clear preference of the FKBD1 for the 3,5 phosphorylated lipid and suggest that the three FKBDs possess a differential affinity for the two PIPs (Figure 3).

The fact that ROF1 bound to the PIPs in the absence of the FKBD1 known to contain the PPIase activity of ROF1 indicates that the presence of an active PPIase domain is not necessary for this interaction as previously suggested for the IPBP12 [40], [41].

Interaction with PI(3)P and PI(3,5)P_2_, phospholipids related to osmotic stress responses [11], [13], as well as the observed enrichment in the chromatography obtained from the NaCl treated culture, pointed to a possible involvement of ROF1 in osmotic stress signaling in plants. We therefore tried to test the possibility of a potential involvement of ROF1 to osmotic stress responses through its PIP binding capacity. Previous studies have demonstrated that ROF1 mRNA progressively accumulates at increasing NaCl concentrations [36]. ROF1 and ROF2 mRNA levels increase in *A. thaliana* roots within 30 mins following 140 mM NaCl treatment [42]. Using Real time PCR mRNA accumulation in response to osmotic/salinity stress was assessed in young leaves of NaCl treated plants and newly germinated seedlings on high osmoticum media. Both ROF1 and ROF2 immediately responded to high salt concentrations at a transcriptional level and their level of response was dose dependent (Figure 5A). mRNA accumulation was also observed in plants grown on high osmoticum media (Figure 5B). Interestingly, this accumulation was observed for the ROF1 mRNA only. Using anti-ROF1 a moderate accumulation of ROF1 at a protein level following salinity stress was demonstrated (Figure 5C). These results are in agreement with the mRNA accumulation pattern produced by the Q-PCR method. Although anti-ROF1 may cross-react to a low extent with ROF2 (Figure 4), no protein accumulation of ROF2 was observed in the case of salt stress (Figure 5C, D). However, it must be noted that even in the case of short term treatments where ROF2 mRNA accumulates (Figure 5A), this accumulation is by no means comparable to the mRNA accumulation observed following heat stress (an 168 fold increase was observed; results not shown).

Localization studies using GFP-tagged ROF1 agreed with previous results [37] suggesting cytoplasmic localization of the protein under non-stress conditions (Figure 6). In order to investigate salinity stress responses of ROF1, immunofluorescence microscopy of root tips of young *A. thaliana* seedlings aiming at the detection of the endogenous protein was performed. Endogenous ROF1 was mainly localized in the elongation zone of the root tip (Figure 7A) a distinct region proposed to generate embryo elongation during the early phase of germination [43]. Short term salt treatment did not alter the pattern of ROF1 localization, however, in seedlings grown on high salt media this localization was diffused throughout the meristematic zone (Figure 7C). The cytoplasmic localization of ROF1 was also confirmed for the endogenous protein (Figure 7A), however, a membrane association of the protein was observed in seedlings germinated on high salt media (Figure 7C). This association supports the documented interaction of ROF1 with PIPs since a biological significance of the latter could imply the recruitment of a soluble, cytoplasmic protein to membrane structures.

ROF1 association with osmotic stress related PIPs, its osmotic stress response patterns as well as its abundance in imbibing seeds and localization in the elongation zone provided the basis for testing its importance in germination under osmotic/ionic stress. Both ROF1 knock out as well as ROF1/2 double knock out seeds were used since the two proteins were co-purified by the PIP chromatography and they have been previously characterized as interacting partners [38]. Germination of *rof1^−^* and *rof2^−^* plants under salt stress was delayed compared to the wild type plants independently of the background ecotype whereas germination was abolished in the WS*rof1^−^/2^−^* double mutants (Figure 8; Figure S5B). In contrast to ROF1, ROF2 has been previously characterized as a bona fide heat stress responsive gene, however, our work suggests its involvement in salt stress tolerance since its absence significantly reduces and further abrogates germination efficiency under salinity stress in the *rof1^−^/2^−^* double mutant (Figure 8). Unlike ROF1, ROF2 mRNA and protein do not accumulate-at least not to a detectable by our antibody level- neither under control (also shown by previous studies) nor under salinity stress (as it occurs during heat stress) (Fig 5). ROF2 became detectable only following its enrichment specifically on the PI(3,5)P_2_ column as demonstrated by our mass spectrometry data, pointing to its specific involvement in a PIP associated osmotic stress related mechanism. The involvement of such a mechanism in osmotic/salinity stress responses was supported by experiments with the PI3K inhibitors wortmannin and LY294002 (Figure 9). Our results point to a PI3K related mechanism involved in seed germination under salinity stress through a ROF1 function. Seeds over-expressing ROF1 showed better germination efficiency in the presence of the inhibitors under normal conditions compared to the knock out plants. In contrast and under salt stress conditions and inhibitor addition, ROF1 overexpression did not enhance seed germination (as it happens in the absence of the inhibitor) and the seeds showed a phenotype similar or identical (in the case of wortmannin) to the double knock out plants during germination.

Though more than one mechanism participate in the regulation of seed germination under osmotic/salinity stress through the function of multi-domain proteins such as ROF1 and ROF2 our experimental evidence suggests that PIP related trafficking events may be of particular importance e.g. by directing chaperonins to a trafficking pathway for the quality control/degradation of misfolded proteins.

Our results show for the first time an association of a FKBP domain containing protein with PIP stereoisomers and suggest a physiological importance of this association in plant responses to osmotic and salt stress.

## Materials and Methods

### PIP Chromatography

PIP chromatography [44] was performed essentially as previously described [45].

*A. thaliana* cell suspension (T87) [46] extract was prepared as follows: For cytosol extraction TNEE buffer containing 50 mM Tris, 80 mM NaCl, 2 mM EGTA, 1 mM EDTA, 2 mM DTT, 300 mM sucrose, protease inhibitor coctail and 1% PVPP was used. Samples were filtered through miracloth and then centrifuged at 10000 g for 15 mins. Pellets were discarded and the supernatant was ultracentrifuged at 50000 g for 1 hr. The supernatant was incubated with Q or S Sepharose (ion exchange chromatography) (Amersham Biosciences, Uppsala, Sweden) for 1 hr. The first flow-through (FT) was collected. In some cases the FT of the Q incubation was collected, directly incubated with the S sepharose and a second flow-through was collected (Q−>S). The Q and S columns were washed successively with buffers of different NaCl concentrations (0.1, 0.2, 0.4, 0.6 and 1 M, pH 6 for S and pH 7.2 for Q). Fractions were concentrated using protein concentrating devices (Centricon, Millipore, UK). Following adjustment of protein concentration using a Bradford based assay (Bio-rad, US) the Q and S fractions were incubated with the phospholipids covalently bound to agarose (Affi-Gel) beads. Beads were washed with 50 mM Tris/0.01% NP40 buffer and proteins were eluted with Laemmli sample buffer containing 20 mM Tris-HCl pH 6.8, 2% SDS, 10% glycerol, 0.1 M DTT and 0.1% Bromophenol blue. Equal volumes were loaded onto SDS-PAGE. Gels were stained with silver nitrate (BDH, UK). For the MS sequencing purposes the gel was stained with colloidal Coomasie (Biorad, UK).

### Mass Spectrometry

PIP binding candidates were identified using nanoLC ESI-MS/MS [47]. The PI(3)P and PI(3,5)P_2_ incubations of the 0.1S fractions were run on a denatured polyacrylamide gel and stained with colloidal Coomasie (Biorad, UK). Bands of interest were excised, reduced, carbamidomethylated, digested with trypsin and analysed by on-line nanoLC ESI-MS/MS.

Tandem mass spectra were extracted, charge state deconvoluted and deisotoped by Mascot.dll version 1.6b25, and searched against the 50961 *A. thaliana* entries in Uniprot release 2011.01, using Mascot version 2.3.02 (Matrix Science, London, UK). The search parameters used specified trypsin as the digestion enzyme, with one missed cleavage allowed: a fragment ion mass tolerance of 0.15 Da and a parent ion tolerance of 150 ppm; carbamidomethylation of cysteine as a fixed modification; cyclisation of peptide N-terminal glutamine and oxidation of methionine, as variable modifications. Scaffold (version 3_00_01, Proteome Software Inc., Portland, USA) was used to validate MS/MS based peptide and protein identifications. Protein identifications were accepted if they could be established at greater than 95.0% probability and contained at least 2 peptides each identified at greater than 50.0% probability.

### ROF1 Cloning

A ROF1 construct in the pVAV44 vector (gift from Prof. Gasser) was used as a template for downstream applications. For subcloning in suitable vectors, primers described in Table S1 were used, and the PCRs were performed using high Fidelity DNA polymerase (Phusion), (Finnzymes-UK). PCR products were first cloned using a pCRII-TOPO TA cloning kit (Invitrogen-UK), transformed into NEB 10-beta *E. coli* cells and sequenced prior to any further use. Ligation for further sub-cloning was performed using T4 DNA ligase (Roche, Germany).

### Protein Overexpression

Identified candidates and their mutants were sub-cloned in the pALEX expression vector for protein overexpression [48]. ROF1 and its truncated mutants were sub-cloned in the indicated sites (Figure 2; Figure S1; Table S1; Table S2). For the FK3TPR construct a direct cloning strategy was followed since digestion of the pVAV44 construct at the Xho1 site produced an in frame insert for cloning in the pALEX vector. The constructs were introduced in BL21 cells [46] -provided by Prof. Sanchez-Serrano - and induction was performed at 28°C, starting when the OD_600_ reached 0.5, for 6 hrs using 0.75 mM IPTG. Bacterial pellets were collected and lysed using 200 µg/ml lysozyme in Tris or PBS buffer. Proteins were purified after two sequential incubations with GST and His columns (Amersham-Pharmacia, Germany) according to the manufacturer’s instructions, collected at the end of the second (nickel column) elution, aliquoted, snap frozen in liquid nitrogen and stored at −80°C in the presence of 10% glycerol. Full length expression of the GST/His tagged proteins was confirmed using the αντι-GST (rabbit polyclonal, Upstate Lake Placid, NY) and the αντι-His mouse monoclonal IgG2a (Amersham Biosciences, Germany).

### Lipid Overlay Assay (Dot Blotting)

A lipid overlay assay was used for the detection of direct protein-lipid interaction. PI, PI(3,4)P_2_, PI(3,5)P_2_, PI(4,5)P_2_ and PI(3,4,5)P_3_ lipids were purchased from Avanti (Alabaster, USA) and PI(3)P, PI(4)P and PI(5)P from Cayman (Ann Arbor, USA). Lipids were diluted in chloroform/methanol and 1 µl (of serial dilutions) of each was spotted on a nitrocellulose membrane (porablot NCL, US). After drying for 30 mins the membrane was incubated with 1% fat free-ultrapure BSA for 1 hr in TBS (50 mM Tris/HCl, 150 mM NaCl and 0,1% tween 20 pH 7,4) for 1 hr at room temperature. Incubation was performed overnight at 4°C using 2 µg/ml purified GST tagged protein. GST-tagged proteins bound to the lipids were detected using αντι-GST. For the lipid competition assay, the same procedure was followed with the additional step of the competing lipid inclusion at a final concentration of 50 µM during the incubation procedure with 1 µg/ml purified GST tagged protein. Equal volume of chloroform/methanol was added in the control (no competing lipid addition) reaction.

### ROF1 Antibody Preparation and Western Blotting

A rabbit polyclonal αντι-ROF1 was prepared and used in Western blotting, dot blotting and immunofluorescence microscopy. Αντι-ROF1 polyclonal antibody was prepared using bacteria overexpressed and purified ROF1. Digestion with factor Xa and Enterokinase (Invitrogen, UK) was performed according to the manufacturer’s instructions. Following digestion the entire amount of the collected protein was run on a polyacrylamide gel under denaturing conditions. Following Coomassie blue staining a clear band of the expected molecular size was cut from the gel and ROF1 was eluted from the polyacrylamide gel using the electroeluter 422 system (Biorad, UK). Rabbits were immunized with 300 µg denatured protein and each immunization was performed in three week intervals. Serum before and after immunization was collected. For cytochemical applications the third bleed was used. Western blotting was performed following SDS-PAGE and transferring to a nitrocellulose membrane. Secondary antibodies goat anti-rabbit and goat anti-mouse horseradish peroxidase-linked for the ECL system were from Chemicon (USA).

### Real time PCR and Protein Detection of Salt/osmotically Stressed Plants

Quanitative, Real Time PCR was performed as previously described [49], [50] in a RG6000 (Corbett Research Pvt Ltd., Sydney, Australia) real time PCR system. Briefly, for short osmotic stress treatments, RNA was extracted from young leaves using the RNA extraction kit (see above) following treatment of 3-week old plants. Either water (control) or water solutions containing different NaCl concentrations were added to the point of overflow and samples from all treatments were collected at different time points. For plants germinated on media of different osmoticum concentrations, emerged seedlings were selected. Seedlings grown on high osmoticum media were of same size as the control seedlings and not of same sowing date since a significant delay in seed germination is recorded under osmotic stress conditions (3 days before for the 0.15 M NaCl treatment and 4–5 days before for the 0.4M mannitol and 0.2 M NaCl treatment). No significant differences in ROF protein/mRNA levels were recorded between 4 to 9 day old seedlings germinated on normal MS media (results not shown). Their RNA was extracted, cDNA was prepared and Q-PCR was performed according to the manufacturer’s instructions using the Platinum Quantitative PCR SuperMix-UDG, SYTO9 (Invitrogen, USA) and the following primers: ROF1 forward: 5′CACCAATGTGAAGGCCTTATACC3; ROF1 reverse: 5′TCAAGACCTCAGGTGCTCATTGC3’; ROF2 forward: 5′TAGGAACGTGAAGGCAATGTATAG3’; ROF2 reverse: 5′TCAATACTCATCGCTTGTGCTTCC3’ UBQ7 forward: 5′GAAGGCATTCCACCTGACCAAC3’ UBQ7 reverse: 5′CAAGCACAAGAAGAAGAAGGTCAAG 3′.

ROF1 and ROF2 fragment sizes were 255 bps and for the Ubiquitin 192 bps. Thermocycler conditions for all primer pairs were as follows: 50°C for 2 mins, 95°C for 2 mins, 36 cycles of 95°C for 5 secs and 60°C for 40 secs. Melting curve was performed from 70–90°C with reading every 0.2°C and 10 secs hold between reads. Quantitation of gene expression was performed [51]. Relative gene expression was estimated using Ubiquitin [49] as an internal control and CTs from triplicate reactions were recorded. Product identity was confirmed for each reaction using High Resolution Melting (HRM) by comparing the picks formed to the ones of the cloned and sequenced product [50].

For protein detection following osmotic stress treatment, samples were collected as described for Real Time PCR. In any case, protein was extracted in Laemmli sample buffer and equal protein amount was loaded in each lane. Bromophenol blue was excluded from the sample buffer, in case the protein assay was performed following extraction using sample buffer, and was added before sample loading on the gel. Protein concentration was determined using the SDS/DTT compatible RC DC protein assay system (Biorad, US). Western blotting was performed as described in the previous section.

### Agrobacterium Tumefaciens Mediated Transformation

ROF1 was sub-cloned in the PVKH18En6 binary vector for *A. tumefaciens* mediated transformation [52] and protein expression in tobacco leaves was performed. The ROF1 construct was prepared using the following primers where the Xba and the Sal1 restriction sites were introduced: Forward: 5′CTAGTCTAGAATGGATGCTAATTTCGAGATGCC3′ Reverse: 5′ACGCGTCGACAATTCCTTACTTAGTTTCGCAAACAT3′.

GFP-tagged protein expression was confirmed by western blotting using a rabbit αντι-GFP polyclonal antibody (Chemicon, USA).

### *Arabidopsis thaliana* Root Tip Preparation for Fluorescence Microscopy

Entire seedlings (3–4 days old) were submerged in a Paraformaldehyde (PFA) solution prepared in 50 mM Pipes, 5 mM EGTA, 5 mM MgSO4 pH 7.0 KOH, for 1 hr with vacuum infiltration applied during the first 5 mins of incubation. Following washing in PBS and H_2_O, seedlings (mainly roots) were placed on top of slides (Superfrost plus-Menzel-Glaser, Germany) and allowed to dry overnight before they were kept at −20°C. After removal from −20°C, slides were placed in a dark humid chamber and immediately hydrated with a PBS buffer before applying 2% driselase in PBS and incubate for 45 mins at 37°C. After washing the driselase solution thoroughly, samples were incubated with 10% DMSO, 0.5% NP40 in PBS buffer for 1 hr at room temperature. Following removal of the permebilisation solution, 5% BSA was applied for 1 hr at room temperature followed by 1 hr incubation with αντι-ROF1 (1∶300) in 5% BSA at 37°C. Samples were further incubated overnight at 4°C with the antibody. Following first antibody washing with PBS, incubation was performed in the Cy3 goat anti-rabbit IgG (Jackson Immunoresearch, USA) for 4 hrs at 37°C. Following washing and DAPI (4′,6-diamidino-2-phenylindole) treatment, mounting solution (0.01% moviol in glycerol) was added and samples were observed under a fluorescence or confocal microscope.

### Fluorescence and Confocal Microscopy

Localization studies of GFP-tagged ROF1 as well as immunofluorescence microscopy were performed using fluorescence and Confocal microscopy. Fluorescence microscopy was performed using an Axioskop 40 fluorescence microscope with a A-Plan Varel contrast 40x/0.65 (Zeiss, Germany). Images were captured using a ProgRes digital camera system from Jenoptic (Germany). For confocal microscopy two confocal microscopes were used; Live cell imaging was performed using a laser Scanning confocal Microscope TCS SP5 SN: 5100000134 with an HCX PL APO CS 63.0×1.20 water UV lens. A pinhole 1 and 1.6 zoom were used. Scan speed was 400 Hz. Resolution was 1024×1024 with 8 bits/pixel. Excitation was achieved with a 488 Argon laser for the GFP and a DPSS 561 for the RFP. Emission bandwidth for GFP was 504.0 nm–521.2 nm and the emission bandwidth for RFP 574.2 nm - 620.1 nm. Fixed tissue imaging was performed using an HC X PLAPO CS 40.0×0.85 dry lens. Pinhole was 2, zoom 1.7 and resolution 1024×1024. Excitation was achieved using a 405 nm Diode for DAPI and a 561 DPSS for Cy3 red fluorescence. Emission bandwidth for DAPI was 423 nm –475.6 nm and the emission bandwidth for Cy3 567 nm –619.4 nm. Image analysis was performed using the LAS AF software version 2.1.0 as well as the ImageJ (Java) software. Also, a Zeiss LSM 510 confocal microscope with a EC Plan-Neofluar 40x/1.30 oil DIC M27 objective was used. Scan zoom was 2. Resolution was 1024×1024 with 8 bits/pixel. Excitation was achieved with a 488 Argon laser. Emission filters were the BP 505–530 for GFP and LP 615 for chloroplast autofluorescence.

### Plant Material and Transformed Lines

Columbia wild type as well as the *rof1^−^* heterozygous plants on a Columbia background, salk_003069, Sail_276_E11 and WiscDsLox502D06 of the Wisconsin DsLox T-DNA lines, were obtained from NASC. Following screening, seeds obtained from the second generation were used in downstream applications. *rof1^−^*, *rof2^−^*, *rof1* ***^−^*** */2^−^*, ROF1OE, and *rof1^−^* complemented mutants on a Wassilevskija background as well as Wassilevskija wild type plants were also used and were a gift from Prof. Adina Breiman [24], [37], [38].

### Germination Rate Under Different Osmotic Stress Conditions

*A. thaliana* seeds of the wild type and mutant lines were disinfected with 70% ethanol for 2 mins and 10% sodium hypochloride for 5 mins. Following a 4–5 day treatment at 4°C to break dormancy, they were seeded on 0.8% agar plates with 1x MS/vitamins containing –where required- NaCl or mannitol. For each characterized line 230–270 seeds were spread on a petri dish. Germination kinetics was determined by recording radicle emergence time after transfer from the stratification conditions to the growth chamber. Plates were scanned in an HP scanjet 4800 series and measurements were performed on the scanned images or under a stereoscope.

### Germination Rate Under Different Osmotic Stress Conditions in the Presence or Absence of PI3K Inhibitors

Germination percentage of WSROF1OE and WS*rof1^−^/2^−^* seeds was recorded at different time points. Following stratification, seeds were germinated in 24 well plates on whatman paper, in water, under dark conditions, at 22°C in the presence or absence of 0.1 M NaCl and in the presence or absence of 33 µM wortmannin (Calbiochem, Germany) or 60 µM LY294002 (Calbiochem, Germany) dissolved in DMSO. An equivalent amount of DMSO was added in the case of the inhibitor absence. The experiment was performed in replicates so that at the end of each time point seeds were collected, scanned and their germination percentage was assessed. The entire experiment was repeated 4 times.

## Supporting Information

Figure S1 **ROF1 domain organisation.** Organisation of the FKBDs, the TPR and the calmodulin binding domain on the amino-acid sequence of ROF1. The polylysine (KKKLLK) and the DSSRDR motives are indicated.
(PDF)

Figure S2 **Structural alignment of ROF1**-β-**Spectrin.** Structural alignment of FKBP12 with β-spectrin, and with the FKBD1, FKBD2 and FKBD3 domains of ROF1. Blue highlight: residues where the I(1,4,5)P_3_ binds to β-spectrin (Hyvonen et al., 1995) or residues on the FKBP12 and the FKBD domains of ROF1 predicted with the potential for inositide binding. DSSP: DSSP algorithm for assigning aminoacid secondary structure [Kabsch, W., and Sander, C. (1983). Dictionary of protein secondary structure: pattern recognition of hydrogen-bonded and geometrical features. Biopolymers *22*, 2577–2637]. L: loop; H: α-helix; E: extended β- structure.
(PDF)

Figure S3 **Anti-ROF1 characterization.** (A) two different serum extractions (1^st^ and 3^rd^) were used to detect the ROF1 overexpressed protein (1) and its truncated mutants 3FKTPR (2), 2FKTPR (3) and TPR (4) (see Figure 2). anti-GST was used to confirm the full length expression of the proteins. (B) Detailed characterization of the specificity of anti-ROF1 for the N-terminus of the protein. anti-ROF1 was used to detect the ROF1 overexpressed protein (1), its truncated mutants 3FK (2), 3FKTPR (3) and the TKFD5 (4) and TKFD7 (5) derived from two different expression lines. (C) anti-ROF1 was used to detect ROF1 overexpressed protein (1) and its truncated mutant NROF1 (2).
(PDF)

Figure S4 **WT and mutant characterization using anti-ROF1.** Characterization of individual lines of *A. thaliana* wild type (Columbia and WS), knock out (SALK, SAIL, WDsLox and WS*rof1^−^*), WSROF1CM and WSROF1OE.
(PDF)

Figure S5 **Germination rate of WT and mutant lines.** (A) Germination rate of the Columbia background (Col-WT) and ROF1 knock out (Col-*rof1^−^*) plants grown on MS medium, B) on MS medium containing 0.2 M NaCl, C) on MS media containing 0.4 M mannitol. D) Germination rate of the Wassilevskija background plants WS, WS*rof1^−^*,WS*rof1^−^*,WS*rof1^−^/2^−^*, WSROF1CM and WSROF1OE on MS medium. Y axis: % germination; X axis: time (hours). Three independent characterized lines (in each experiment) were used for each genotype. Results are average of three independent experiments performed for each treatment. Values are means and bars are SDs.
(PDF)

Table S1 **Primers used for the introduction of restriction sites (highlighted) and subsequent cloning.**
(PDF)

Table S2 **Constructs of ROF1 and of its truncated mutants.**
(PDF)

Movie S1 **Plasmolysis effect on ROF1-GFP localization following 0.25 M NaCl.**
(AVI)

Movie S2 **Endogenous ROF1 localization using anti-ROF1– control.**
(AVI)

Movie S3 **Endogenous ROF1 localisation using anti-ROF1–0.2 M NaCl.**
(AVI)

Spectrum Report S1 (XLSX)
