# Supplementary material for: The Arabidopsis thaliana Immunophilin ROF1 Directly Interacts with PI(3)P and PI(3,5)P2 and Affects Germination under Osmotic Stress
Source: PLoS One. 2012 Nov 2;7(11):e48241. doi: 10.1371/journal.pone.0048241 (PMC3487907; doi:10.1371/journal.pone.0048241)
Supplement: Figure S1 — ROF1 domain organisation. Organisation of the FKBDs, the TPR and the calmodulin binding domain on the amino-acid sequence of ROF1. The polylysine (KKKLLK) and the DSSRDR motives are indicated. (PDF) [file pone.0048241.s001.pdf]

FIGURE S1

```
        Low Complexity Region
-------------------------------------|------------
1         10        20        30        40
|....|....|....|....|....|....|....|....|....|....
MDANFEMPPVGGMNDDDDMDFGDGASFLKVGEEKEIQQGLKKKLLKEGEG

                     FKBD1
--------------------------------------------------
50        60        70        80        90
|....|....|....|....|....|....|....|....|....|....
YETPENGDEVEVHYTGTLLDGTKFDSSRDRATPFKFTLGQGQVIKGWDIG

--------------------------------------------|
100       110       120       130       140
|....|....|....|....|....|....|....|....|....|....
IKTMKKGENAVFTIPAELAYGESGSPPTIPANATLQFDVELLKWDSVKDI

             |------------------------------------
150       160       170       180       190
|....|....|....|....|....|....|....|....|....|....
CKDGGVFKKILAVGEKWENPKDLDEVLVKFEAKLEDGTVVGKSDGVEFTV

                      FKBD2
--------------------------------------------------
200       210       220       230       240
|....|....|....|....|....|....|....|....|....|....
KDGHFCPALTKAVKTMKKGEKVLLTVKPQYGFGEKGKPASAGEGAVPPNA

-----------|            |-------------------------
250       260       270       280       290
|....|....|....|....|....|....|....|....|....|....
TLEINLELVSWKTVSEVTDDNKVVKKVLKEGDGYERPNEGAVVKVKLIGK

                       FKBD3
--------------------------------------------------
300       310       320       330       340
|....|....|....|....|....|....|....|....|....|....
LQDGTVFLKKGHGENEEPFEFKTDEEQVVDGLDRAVMKMKKGEVALVTID

---------------------------------|
350       360       370       380       390
|....|....|....|....|....|....|....|....|....|....
PEYAFGSNESQQELAVVPPNSTVTYEVDLLTFDKERESWDMNTEEKIEAA

                TPR1
  |---------------------------------|
400       410       420       430       440
|....|....|....|....|....|....|....|....|....|....
SKKKEEGNSKFKGGKYSLASKRYEKAVKFIEYDTSFSEEEKKQAKALKVA

              TPR2                         TPR3
|---------------------------------||--------------
450       460       470       480       490
|....|....|....|....|....|....|....|....|....|....
CNLNDAACKLKLKDYKQAEKLCTKVLELESTNVKALYRRAQAYMELSDLD

                                      CaMBD
-------------------|        |---------------|
500       510       520       530       540       550
|....|....|....|....|....|....|....|....|....|....|.
LAEFDVKKALEIDPNNREVKLEQKRLKEKMKEFNKKEAKFYGNMFAKLSKE.
```
