# Supplementary material for: The Arabidopsis thaliana Immunophilin ROF1 Directly Interacts with PI(3)P and PI(3,5)P2 and Affects Germination under Osmotic Stress
Source: PLoS One. 2012 Nov 2;7(11):e48241. doi: 10.1371/journal.pone.0048241 (PMC3487907; doi:10.1371/journal.pone.0048241)
Supplement: Figure S2 — Structural alignment of ROF1-β-Spectrin. Structural alignment of FKBP12 with β-spectrin, and with the FKBD1, FKBD2 and FKBD3 domains of ROF1. Blue highlight: residues where the I(1,4,5)P3 binds to β-spectrin (Hyvonen et al., 1995) or residues on the FKBP12 and the FKBD domains of ROF1 predicted with the potential for inositide binding. DSSP: DSSP algorithm for assigning aminoacid secondary structure [Kabsch, W., and Sander, C. (1983). Dictionary of protein secondary structure: pattern recognition of hydrogen-bonded and geometrical features. Biopolymers 22, 2577–2637]. L: loop; H: α-helix; E: extended β- structure. (PDF) [file pone.0048241.s002.pdf]

# FIGURE S2

## FKBP12-FKBD1-SPECTRIN

```
DSSP     ..........LEEEEEEELLLLLLLLLLLLEEEEEEEEEEL..LLLE.EEELLLLLLLEEEEL
FKBP12   ..........GVQVETISPGDGRTFPKRGQTCVVHYTGMLE..DGKK.FDSSRDRNKPFKFML
ident              |        | |   |  |    ||||| |   || | |||||||  |||| |
FKBD1    kvgeekeiqqGLKKKLLKEGEGYETPENGDEVEVHYTGTLL..DGTK.FDSSRDRATPFKFTL
DSSP     leelllllllLLEEEEEELLLLLLLLLLLLEEEEEEEEEEL..LLLE.EEEHHHHLLLEEEEL
ident                                            |    |    |
DSSP                                  LEEEEEEEEEELllLLLLlLLLLE....EEEEEE
spectrin                              MEGFLNRKHEWEahNKKAsSRSWH....NVYCVI
ident                                            |    |    |
FKBP12   ..........GVQVETISPGDGRTFPKRGQTCVVHYTGMLE..DGKK.FDSSRDRNKPFKFML


DSSP     LLL....................LLLHH....HHHHHHLLLLLLEEEEEELHHHLLLLLLLLL
FKBP12   GKQ....................EVIRG....WEEGVAQMSVGQRAKLTISPDYAYGATGHPG
ident    |                       || |    |  |   |  |  |  ||    |||  | |
FKBD1    GQG....................QVIKG....WDIGIKTMKKGENAVFTIPAELAYGESGSPP
DSSP     LLL....................LLLHH....HHHHHLLLLLLLEEEEEELHHHLLLLLLLLL
ident      |                                              ||  |
DSSP     ELLeeeeellhhhhhhllllllllLEELllleeeelllllllLLEEEEELLL...........
spectrin NNQemgfykdaksaasgipyhsevPVSLkeaicevaldykkkKHVFKLRLSD...........
ident      |                                              ||  |
FKBP12   GKQ....................EVIRG....WEEGVAQMSVGQRAKLTISPDYAYGATGHPG


DSSP     LLLLLLLEEEEEEEE............EEL.
FKBP12   IIPPHATLVFDVELL............KLE.  107
ident     ||  ||| ||||||            |
FKBD1    TIPANATLQFDVELL............KWDs  118
DSSP     LLLLLLLEEEEEEEE............EEEl
ident
DSSP     ......LLEEEEELLLlhhhhhhhhhhhhhl
Spectrin ......GNEYLFQAKddeemntwiqaissa
ident
FKBP12   IIPPHATLVFDVELL............KLE
```

## FKBD2-SPECTRIN

```
DSSP     leellllLLEEEEEEELLL..........llllllLLLE.....................
FKBD2    vkdickdGGVFKKILAVGE..........kwenpkDLDE..................... 29
ident           |    |                         |
spectrin .....meGFLNRKHEWEAHnkkassrswhnvycviNNQEmgfykdaksaasgipyhsevp 55
DSSP     .....leEEEEEEEEELLLllllllllllleeeeeeeELLEeeeellhhhhhhllllllll


DSSP     ...EEEEEEEELllllleeeeeeeeeeelllllllllhhhhllllLLLEEEEEELH.HHL
FKBD2    ...VLVKFEAKLedgtvvgksdgveftvkdghfcpaltkavktmkKGEKVLLTVKP.QYG 85
ident            |  |                                  |   |   |
spectrin vslKEAICEVAL...................dykkkkhvfklrlsDGNEYLFQAKDdEEM 96
DSSP     eelLLLEEEELL...................llllllleeeeellLLLEEEEELLLhHHH


DSSP     Llllllhhhlll
FKBD2    Fgekgkpasage   97
ident
spectrin N..twiqaissa  106
DSSP     H..hhhhhhhhl
```

## FKBD3-SPECTRIN

```
DSSP      lllllllleeeeeeellllllllllllLEEEEEEEEEEEellllEEEEEE.EEEEEEELL
FKBD3     vsevtddnkvvkkvlkegdgyerpnegAVVKVKLIGKLQdgtvfLKKGHG.ENEEPFEFK 59
ident                                                    |
spectrin  ...........................MEGFLNRKHEWE..ahnKKASSRsWHNVYCVIN 31
DSSP      ...........................LEEEEEEEEEEL..lllLLLLLLlLEEEEEEEE


DSSP     ...............lLLLL...lLHHH....hhhhlllllLEEEEEEELHhhllhhhll
FKBD3    ...............tDEEQ...vVDGL....dravmkmkkGEVALVTIDPeyafgsnes 97
ident                                    |                  |
spectrin nqemgfykdaksaasgIPYHsevpVSLKeaicevaldykkkKHVFKLRLSD......... 82
DSSP     lleeeeellhhhhhhlLLLLllllEELLlleeeelllllllLLEEEEEELL.........


DSSP     lllllllllllLEEEEEEEE......eeellllll
FKBD3    qqelavvppnsTVTYEVDLL......tfdkeresw  126
ident                  |
spectrin ...........GNEYLFQAKddeemntwiqaissa  106
DSSP     ...........LLEEEEELLlhhhhhhhhhhhhhl
```
