# Supplementary figures and images for: The Arabidopsis thaliana Immunophilin ROF1 Directly Interacts with PI(3)P and PI(3,5)P2 and Affects Germination under Osmotic Stress
Source: PLoS One. 2012 Nov 2;7(11):e48241. doi: 10.1371/journal.pone.0048241 (PMC3487907; doi:10.1371/journal.pone.0048241)

**FIGURE S3A**

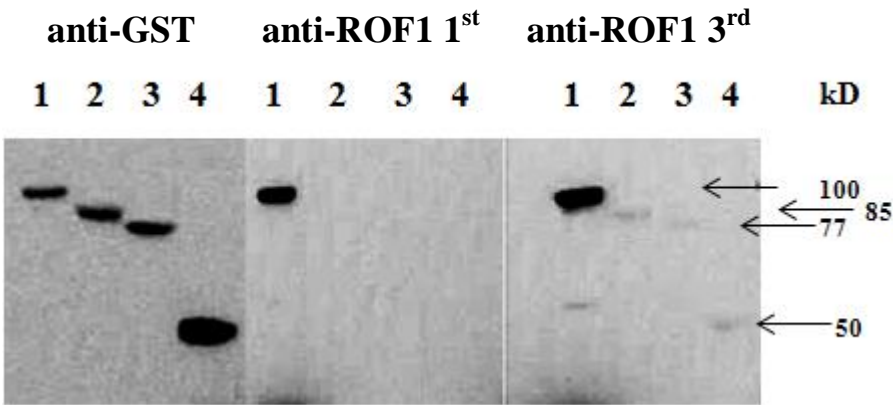

**FIGURE S3B**

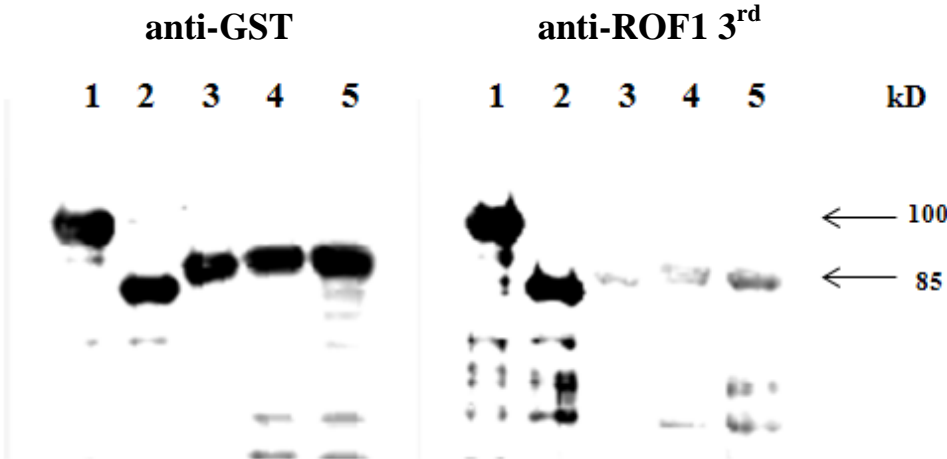

**FIGURE S3C**

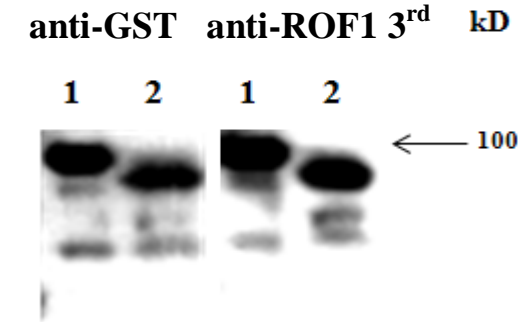

Supplement: Figure S3 — Anti-ROF1 characterization. (A) two different serum extractions (1st and 3rd) were used to detect the ROF1 overexpressed protein (1) and its truncated mutants 3FKTPR (2), 2FKTPR (3) and TPR (4) (see Figure 2). anti-GST was used to confirm the full length expression of the proteins. (B) Detailed characterization of the specificity of anti-ROF1 for the N-terminus of the protein. anti-ROF1 was used to detect the ROF1 overexpressed protein (1), its truncated mutants 3FK (2), 3FKTPR (3) and the TKFD5 (4) and TKFD7 (5) derived from two different expression lines. (C) anti-ROF1 was used to detect ROF1 overexpressed protein (1) and its truncated mutant NROF1 (2). (PDF) [file pone.0048241.s003.pdf]

**FIGURE S4**

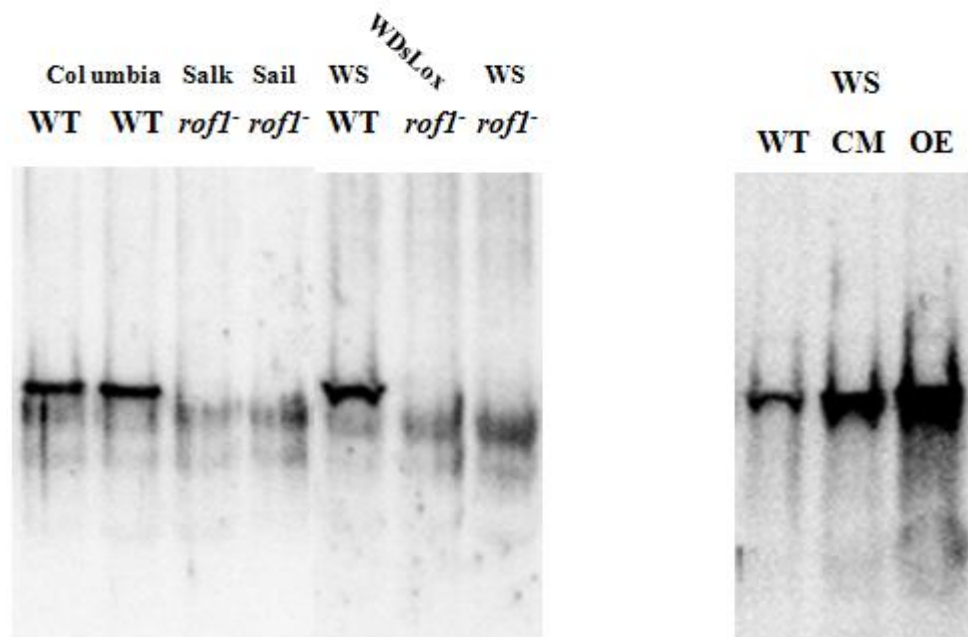

Supplement: Figure S4 — WT and mutant characterization using anti-ROF1. Characterization of individual lines of A. thaliana wild type (Columbia and WS), knock out (SALK, SAIL, WDsLox and WSrof1−), WSROF1CM and WSROF1OE. (PDF) [file pone.0048241.s004.pdf]

**FIGURE S5A**

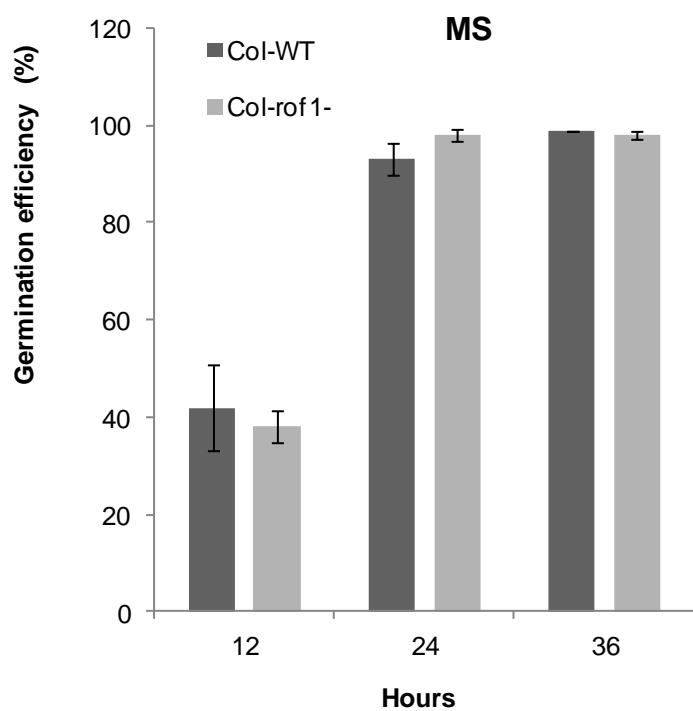

**FIGURE S5B**

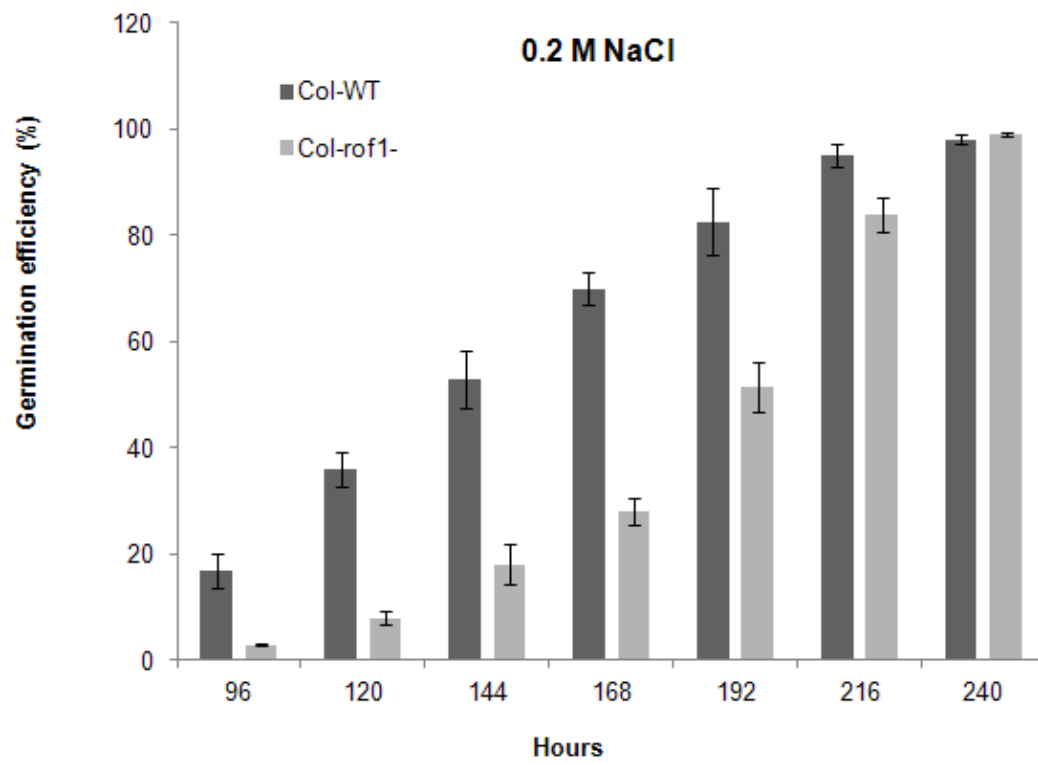

**FIGURE S5C**

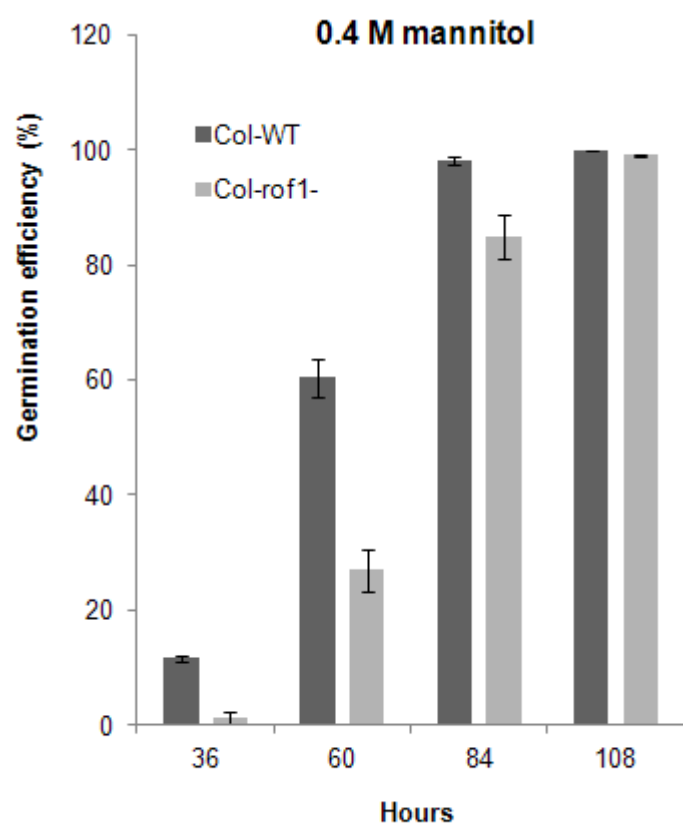

**FIGURE S5D**

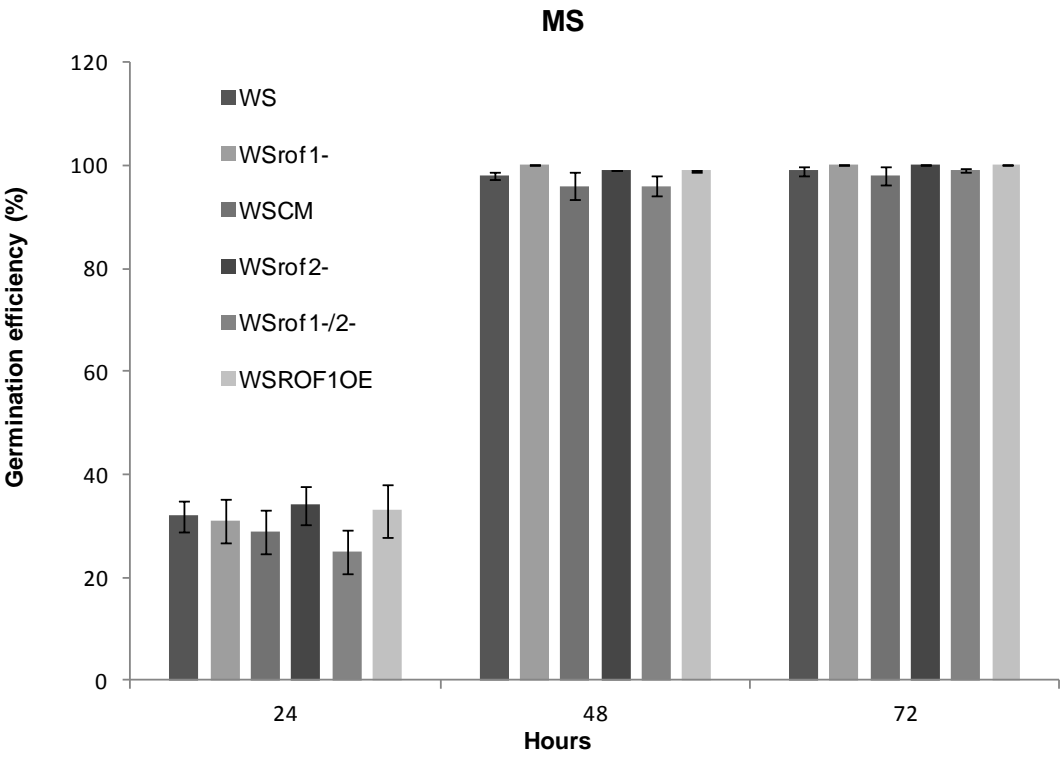

Supplement: Figure S5 — Germination rate of WT and mutant lines. (A) Germination rate of the Columbia background (Col-WT) and ROF1 knock out (Col-rof1−) plants grown on MS medium, B) on MS medium containing 0.2 M NaCl, C) on MS media containing 0.4 M mannitol. D) Germination rate of the Wassilevskija background plants WS, WSrof1−,WSrof1−,WSrof1−/2−, WSROF1CM and WSROF1OE on MS medium. Y axis: % germination; X axis: time (hours). Three independent characterized lines (in each experiment) were used for each genotype. Results are average of three independent experiments performed for each treatment. Values are means and bars are SDs. (PDF) [file pone.0048241.s005.pdf]
