# Supplementary material for: The Arabidopsis thaliana Immunophilin ROF1 Directly Interacts with PI(3)P and PI(3,5)P2 and Affects Germination under Osmotic Stress
Source: PLoS One. 2012 Nov 2;7(11):e48241. doi: 10.1371/journal.pone.0048241 (PMC3487907; doi:10.1371/journal.pone.0048241)
Supplement: Table S1 — Primers used for the introduction of restriction sites (highlighted) and subsequent cloning. (PDF) [file pone.0048241.s006.pdf]

TABLE S1

PRIMER	SEQUENCE	CLONING SITE	VECTOR CLONED
	**Protein overexpression in bacterial cells**		
FKBPF	GGGGATCCCCATGGATGCTAATTTCGAGAT	BAMH1/NCO1	PALEX
FKBPR	GAGTGCGGCCGCACTTAGTTTCGCAAACAT	NOT1	PALEX
1FKR	GTGCTCGAGCTTCACACTGTCCCATTTAAGC	XHO1	PALEX
3FKOER	ATAGTTTAGCGGCCGCGTTCATGTCCCACGATTCCCT	NOT1	PALEX
3FKTPRF	GTGGGATCCCTTTCAAATTCACTCTTGGACA	BAMH1	PALEX
2FKTPRF	GTGGGATCCCCAGTGTGAAGGATATATGTAAAG	BAMH1	PALEX
TPRF	GTGGGATCCCCACTGAGGAGAAGATTGAAGCT	BAMH1	PALEX
TKFD	GTGGGATCCCGACGAAGTTTGATTCGAGTCGTGA	BAMH1	PALEX
NROF1	GTGGGATCCCAAGGTTTGAAGAAGAAGCTTCTT	BAMH1	PALEX
