# Supplementary material for: The Arabidopsis thaliana Immunophilin ROF1 Directly Interacts with PI(3)P and PI(3,5)P2 and Affects Germination under Osmotic Stress
Source: PLoS One. 2012 Nov 2;7(11):e48241. doi: 10.1371/journal.pone.0048241 (PMC3487907; doi:10.1371/journal.pone.0048241)
Supplement: Table S2 — Constructs of ROF1 and of its truncated mutants. (PDF) [file pone.0048241.s007.pdf]

TABLE S2

PRIMERS	CONSTRUCT NAME	VECTOR CLONED
	**ROF1 AND ROF1 TRUNCATED MUTANT FUSIONS FOR PROTEIN OVEREXPRESSION**	
FKBPF/FKBPR	ROF1	PALEX
NROF1/FKBPR	NROF1	PALEX
TKFD/FKBPR	TKFD	PALEX
3FKTPRF/FKBPR	3FKTPR	PALEX
2FKTPRF/FKBPR	2FKTPR	PALEX
FKBPF/1FKR	1FK	PALEX
FKBPF/3FKOER	3FK	PALEX
TPRF/FKBPR	TPR	PALEX
direct cloning	FK3TPR	PALEX
